# Immediate versus conditional treatment of uncomplicated urinary tract infection - a randomized-controlled comparative effectiveness study in general practices

**DOI:** 10.1186/1471-2334-12-146

**Published:** 2012-06-28

**Authors:** Ildikó Gágyor, Eva Hummers-Pradier, Michael M Kochen, Guido Schmiemann, Karl Wegscheider, Jutta Bleidorn

**Affiliations:** 1 Department of General Practice and Family Medicine, University of Goettingen, Humboldtallee 38, Goettingen, 37073, Germany; 2 Institute of General Practice, Hannover Medical School, Carl-Neuberg-Str.1, Hannover, 30625, Germany; 3 Institute for Public Health and Nursing Research, Department for Health Services Research, University of Bremen, Grazer Str. 4, Bremen, 28359, Germany; 4 Department of Medical Biometry and Epidemiology, University Medical Centre Hamburg-Eppendorf, Martinistr. 52, Hamburg, 20246, Germany

**Keywords:** Urinary tract infection, Comparative effectiveness design, Antibiotic prescription, General practice

## Abstract

**Background:**

Uncomplicated urinary tract infections (UTI) are usually treated with antibiotics as recommended by primary care guidelines. Antibiotic treatment supports clinical cure in individual patients but also leads to emerging resistance rates in the population. We designed a comparative effectiveness study to investigate whether the use of antibiotics for uncomplicated UTI could be reduced by initial treatment with ibuprofen, reserving antibiotic treatment to patients who return due to ongoing or recurrent symptoms.

**Methods/design:**

This is a randomized-controlled, double-blind, double dummy multicentre trial assessing the comparative effectiveness of immediate vs. conditional antibiotic therapy in uncomplicated UTI. Women > 18 and < 65 years, presenting at general practices with at least one of the typical symptoms dysuria or frequency/urgency of micturition, will be screened and enrolled into the trial. During an 18- months recruitment period, a total of 494 patients will have to be recruited in 45 general practices in Lower Saxony. Participating patients receive either immediate antibiotic therapy with fosfomycin-trometamol 1x3g or initial symptomatic treatment with ibuprofen 3x400mg for 3 days. The ibuprofen group will be provided with antibiotic therapy only if needed, i.e. for persistent or worsening symptoms. For a combined primary endpoint, we choose the number of all antibiotic prescriptions regardless of the medical indication day 0–28 and the “disease burden”, defined as a weighted sum of the daily total symptom scores from day 0 to day 7. The study is considered positive if superiority of conditional antibiotic treatment with respect to the first primary endpoint and non-inferiority of conditional antibiotic treatment with respect to the second primary endpoint is proven.

**Discussion:**

This study aims at investigating whether the use of antibiotics for uncomplicated UTI could be reduced by initial treatment with ibuprofen. The comparative effectiveness design was chosen to prove the effectiveness of two therapeutic strategies instead of the pure drug efficacy.

**Trial registration:**

Clinicaltrials.Gov: NCT01488955

## Background

Uncomplicated urinary tract infections (UTI) are a common condition in general practice. To date, they are usually treated with antibiotics, as recommended by primary care guidelines [1-3] - resulting in effective and fast symptom resolution. However, this approach accounts for a substantial number of antibiotic prescriptions in primary care with inherent disadvantages [4].

The increasing use of antibiotics is the main reason for emerging resistance rates [5,6]. More than 20% of E.coli, the most common uropathogens, are resistant to trimethoprim-sulfomethoxazole and to cephalosporins [7]; for fluoroquinolons the same trend can be observed. Since less antibiotic prescribing is associated with lower levels of antibiotic resistance [8,9], efforts should be made to reduce unnecessary prescriptions [10,11]. Up to now, only few randomized-controlled trials compared antibiotic treatment with placebo or different therapeutic strategies, i.e. empirical, targeted or delayed antibiotic treatment in uncomplicated UTI [12-14]. These trials suggested that in many cases uncomplicated UTI are a self limiting condition. Results showed delayed symptomatic and bacteriological cure in the placebo and in the delayed prescription group, but no serious complications [4,7,12,14]. The failure rate in the placebo group due to symptom persistence/worsening was considered a strong argument in favour of antimicrobial treatment [10]. In 2007/2008, the authors carried out a randomized controlled pilot trial comparing ibuprofen and ciprofloxacin in uncomplicated UTI with regard to symptom course Non-inferiority of ibuprofen could be demonstrated based on a sample size of 79 patients. Symptomatic treatment was sufficient for 66% (24/36) patients in the ibuprofen-group, with secondary antibiotic treatment rates of 33% (12/36) versus 18% (6/33) in the ibuprofen and ciprofloxacin groups respectively [15]. In view of the symptomatic treatment as alternative treatment option for uncomplicated UTI, we designed a comparative effectiveness study with the aim to investigate whether the use of antibiotics for uncomplicated UTI could be reduced by initial treatment with ibuprofen, reserving antibiotic treatment to patients who return due to ongoing or recurrent symptoms.

Our main research questions are: 1) Does conditional antibiotic use in patients with uncomplicated UTI reduce the number of antibiotic prescriptions without significant increase in symptoms or recurrent episodes/complications? 2) Is the proposed strategy safe in terms of complications and recurrences?

## Methods

### Design

This is a randomized-controlled, double-blind, double dummy multicentre trial assessing the comparative effectiveness of immediate vs. conditional antibiotic therapy in uncomplicated UTI.

### Patients

#### Inclusion criteria

Women > 18 and < 65 years with suspected UTI, presenting at general practices with at least one of the typical symptoms dysuria or frequency/urgency of micturition. Written informed consent is required.

Key *exclusion criteria* are any signs of complicated infection (temperature > 38 °C, pain on renal bed percussion; any conditions that may lead to complicated infections (i.e. pregnancy, renal diseases, immunosuppressive therapy); current antibiotic therapy, current intake of NSAIDs, current intake of drugs interacting with the trial drugs (anticoagulants, corticosteroids) contraindications or allergies for trial drugs; renal diseases (renal failure; urinary tract abnormalities or past urinary surgery, urinary catheterization); history of gastrointestinal ulcers; serious neurological diseases (epilepsy, multiple sclerosis, paraplegia); disability to understand trial information.

#### Setting and recruitment

The trial will be carried out in general practices in northern Germany. A total of 494 patients will have to be recruited in 45 investigator sites (general practices) during an 18- months recruitment period. Women presenting with UTI symptoms will be screened and enrolled by their GP.

To achieve the recruitment goal as soon as possible, high emphasis will be put on structured practice support to optimize patient recruitment (i.e. newsletters, telephone calls, incentives).

#### Intervention/trial drug

Participating patients receive either immediate antibiotic therapy with fosfomycin-trometamol 1x3g or initial symptomatic treatment with ibuprofen 3x400mg for 3 days. Antibiotic therapy will be only provided if needed, i.e. for persistent or worsening symptoms.

Fosfomycin for oral treatment is only available as granules. Thus, a double-dummy-design is required with the intervention group taking placebo granule sachets 1x1 additional to 3x400 mg ibuprofen tablets and the control group taking placebo tablets 3x1/3 days additional to fosfomycin-trometamol.

During the entire trial, long term medication or co-medication should be prescribed and taken as usual. Patients will be told to avoid taking spasmolytic drugs. Any further medication will be documented in Case Report Form (CRF). If secondary antibiotic treatment is required due to ongoing/worsening symptoms, antibiotics should be chosen in line with the results of the urine culture at day 0.

In order to guarantee concealment a central randomization on patient level will be performed. Code numbers will be assigned from the random list to the drug units in blocks of six and distributed to the participating practices. At inclusion, the code number will be assigned and the drug package will be transferred to the patient by the GP.

### Procedures

#### Day 0/inclusion

Patients complete a symptom questionnaire and provide a urine specimen for culture, dipstick and pregnancy test. Temperature will be taken oral or auricular. The GP hands over the blinded trial drug and instructs the patient to reconsult in case of persistent or worsening symptoms, or in case of fever. In uncomplicated cases the number of mandatory study visits will be restricted to day 0. If the patient returns within three days with persistent or worsening symptoms of UTI, the study drug will be discontinued and replaced by an antibiotic at the discretion of the GP, chosen in line with urine culture results if available. Recurrent UTI after the end of the study treatment will be treated with antibiotics at the discretion of the GP. All urine cultures will be performed in one central laboratory. Native urine will be transported within 24 h to the laboratory; in the meantime it will be stored in the refrigerator.

### Data collection and management

#### Inclusion

Patients complete a piloted symptom questionnaire to score severity of each symptom (dysuria, frequency and low abdominal pain). Symptoms will be scored from 0 (not at all) to 4 (very strong), and symptom scores will be added to a maximal sum score of 12 points. The symptom questionnaire has already been piloted in the study “HWI-01” in 79 patients. Additionally, patients will record the UTI-related impairment in daily live-activities by the Activity Impairment Assessment (AIA) [16].

Demographic and clinical data and laboratory results will be documented by the practice staff in an internet-based database (eCRF).

#### Follow up

**Figure 1:**
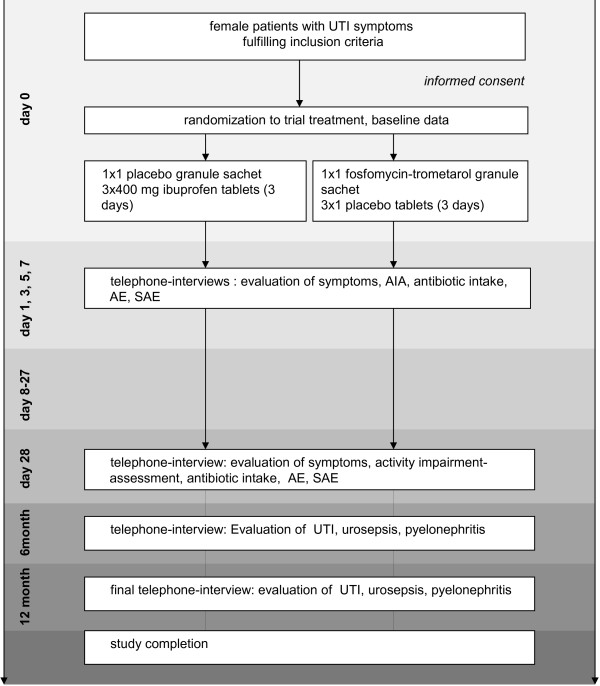
**Study plan.** Abbreviations: UTI= Urinary Tract Infection, AE= Adverse Event, SAE= Serious Adverse Event, AIA= Activity Impairment Assessment.

Further symptom assessment will be conducted via telephone interviews by the study nurses of the University Departments' research teams. Symptom related data and impairment in daily live-activities (AIA) will be scored according to the symptom questionnaire at inclusion. Additionally, cause, duration and dosage of any antibiotic prescriptions will be recorded, as well as adverse events. Telephone interviews will be conducted on day 1, 3, 5 and 7, or until resolution of symptoms. During this time, patients will complete a symptom diary daily to collect continuous data by telephone interviews.) The last telephone interview will take place on day 28. To complete follow-up data in terms of recurrences and safety information, further interviews will be conducted after 6 and 12 months (Figure 1).

### Outcomes

#### Primary endpoints

Two co-primary endpoints measuring number of antibiotic prescriptions and disease burden are considered.

##### First co-primary endpoint

Number of all antibiotic prescriptions regardless of the medical indication day 0–28.

##### Second co-primary endpoint

The “disease burden“will be calculated as weighted sum of the daily total symptom scores from day 0 to day 7. The weights are chosen in a way that the measure represents the area under curve (AUC) below the total symptom score as a function of time.

#### Key secondary endpoints

Sum of daily defined doses (DDD) per patient; proportion of patients with/number of recurrent UTI episodes within 28 days/6/12 months, defined by the re-occurrence of UTI symptoms after symptom resolution; proportion of symptom-free patients at day 4 and 7; total disease burden (AUC) until day 4; disease burden related to each of the three symptoms until day 7; activity impairment assessment (AIA) day 1 to day 7; number of complications (febrile UTI, pyelonephritis/septic syndrome) and the number of adverse events.

The defined daily doses (DDD) are based on the official ATC-Index for Germany 2010 [17], which is derived from the WHO-index 2010 [18].

“Symptom-free” is defined as a total symptom score of 0.

### Statistical analysis and reporting

The primary analysis will be based on the results of two statistical tests. The first test (superiority) will be on the following two hypotheses with respect to the first co-primary endpoint:

H0: The number of antibiotic prescriptions within the interval 0–28 days in the conditional use group is greater than or equal to the corresponding number in the immediate use group.

vs.

H1: The number of antibiotic prescriptions within the interval 0–28 days in the conditional use group is lower than the corresponding number in the immediate use group.

The second test (non-inferiority) will be on the following two hypotheses with respect to the second co-primary endpoint:

H0: The disease burden within the interval 0–7 days in the conditional use group is greater than or equal to 125% of the corresponding disease burden in the immediate use group.

vs.

H1: The disease burden within the interval 0–7 days in the conditional use group is less than 125% of the corresponding disease burden in the immediate use group.

Since both criteria have to be fulfilled for the study being positive, the intersection hypothesis has to be proven. This can effectively be achieved by a closed testing procedure where both hypotheses are tested hierarchically at a level of 2.5% one-sided, i.e. the second hypothesis will only be tested if the first test was significant.

Formally, hypothesis (i) will be tested non-parametrically using an exact Mann–Whitney rank sum test since the distribution of antibiotic prescriptions will be discrete and is not expected to follow a known standard distribution. The test will be performed in the intention-to-treat (ITT) population consisting of all patients randomized with at least one report on antibiotic use. For hypothesis (ii) to be tested, a covariance analysis of the log disease burden will be performed with the random group as factor and the day-0 (inclusion) log sum of symptom scores as covariate. Logarithms were chosen since the pilot study suggested that the distribution of disease burden was approximately normal after this transformation was applied. From this model, a two-sided 95% confidence interval for the ratio of the expected total disease burdens of conditional use vs. immediate use will be calculated by back-transformation (exponentiation) of the corresponding interval in log-units to the original scale. If this interval will lie entirely below 1.25, non-inferiority of conditional use therapy as compared to immediate use therapy can be stated. We pre-defined the margins of equivalence to be 80% to 125% as these margins are understood as minimum clinically relevant deviations. The analysis will be performed in the per protocol (PP) population consisting of all patients randomized with complete symptom score over 7 days and therapy in line with the study protocol. The analysis will be repeated in the ITT population to judge the validity of the per-protocol result.

Missing values will pose a serious challenge to the validity of the analysis since up to 15% premature study terminations due to non-compliance or withdrawal of consent are to be expected. For the ITT analyses, some kind of imputation of missing values in the co-primary endpoints is required. We thus decided to perform two ITT analyses for each endpoint. The primary analysis uses multiple imputations of missing values based on the available information. Last observation carried forward analysis will also be performed as an additional conservative sensitivity analysis. The technical details will be defined in a statistical analysis plan after a blind review of the data, before code break.

Secondary endpoints will be analyzed by Mann–Whitney-U Tests tests (numbers) or analogous to the second co-primary endpoints, i.e. calculation of between-group differences or ratios with 95%-confidence limits, taking the different scale types into account. To characterise patients who will need antibiotic treatment in the conditional use group, an exploratory logistic regression analysis will be performed.

### Sample size

The sample sizes required for the superiority and the non-inferiority part of the trial were based on the results of the pilot study, but were chosen rather conservative. For the superiority part, antibiotic prescription rates of 50% and 90% were assumed for conditional and immediate use, resulting in a sample size of 2x29 = 58 patients to yield a power of 90%. For the non-inferiority part, a coefficient of variation of 80% was assumed for disease burden, resulting in a sample size of 2x210 = 420 patients to reach a power of 90% for the case of zero group difference. Thus, the second hypothesis drives the sample size. With an assumed drop-out rate of 15% for the PP population, 494 patients have to be randomized. Calculations were done using PASS 2008.

### Safety

At inclusion, patients will be instructed to reconsult at any time in case of ongoing/worsening symptoms. For this case, an immediate specific antibiotic therapy can be provided based on the urine culture at inclusion.

Adverse events (AE) leading to practice consultation will be documented by the GP; minor AEs will be actively asked for during telephone interviews. Serious AE and patients reconsulting with fever will have to be reported immediately. An independent Data and Safety Monitoring Board is established to examine safety risks based on the safety related data regularly.

Adverse events rates in both groups serve as safety outcomes and will be compared in data analysis. Late follow ups after 6 and 12 months serve to collect observational data of recurrences and other renal complications.

### Ethical considerations

Ethical approval has been obtained by the Independent Ethics Committees of the Hannover Medical School (No. 5986 M). The study will be conducted according to the principles of Good Clinical Practice.

Informed consent is taken by the GP who also ensures complete information about risks, benefits, and study procedures etc., based on a patient information sheet which is prepared according to current ethic committees standards. Patients can cancel their consent for the trial at any time without disadvantages.

All data including patient identifiers will be treated confidentially. The patient declares her agreement to disclosure of pseudonymised data based on current data protection regulations.

### Registration

This study is registered at clinical trials.gov (Nr. 2011-002271-42) with the acronym ICUTI.

## Discussion

This study aims at investigating whether the use of antibiotics for uncomplicated UTI could be reduced by initial treatment with ibuprofen. The comparative effectiveness design was chosen to prove the effectiveness of two therapeutic strategies instead of the pure drug efficacy. This approach, fitting in the daily routines of UTI management in primary care [19], can eventually alter the way in which treatment of UTI is usually practiced. ICUTI is designed to create evidence for an alternative treatment option without antibiotics and to provide information about risks and benefits of both treatment strategies.

While working out the study design, we considered different methodological approaches, one of which would have been a non-inferiority trial with a placebo-group [20]. This could also have demonstrated the superiority of the other two treatment strategies over placebo, but we decided that these methodological benefits did not warrant withholding available treatments from patients who suffer from acute UTI. Therefore we decided against this approach.

Many UTI trials focus on patients with microbiologically proven UTI. We decided to follow a more pragmatic approach by including patients presenting with typical symptoms. The resulting study population represents “typical” patients with uncomplicated lower UTI who are otherwise healthy.

Urine cultures will only be provided for trial reasons on day 0 to assess bacterial count and species, to be able to discriminate patients with and without bacterial infections in the analysis, and to have data on resistance in case a secondary antibiotic treatment is needed. This is in line with general practitioners’ routine and general practice guidelines – treatment decisions in uncomplicated UTI are usually made without microbiological specification.

### Procedures

To optimize external validity, study-related changes of usual general practice procedures are minimized. Therefore, in line with routine GP procedures in primary care, study visits are restricted to inclusion on day 0. Further study visits are not mandatory. However, patients are instructed to return to see their doctor in case of persistent or recurrent symptoms, or if they wish to consult for other reasons.

### Trial drug

For immediate antibiotic therapy, fosfomycin-trometamol was chosen as first line treatment. The German UTI guideline recommends fosfomycin-trometamol as one of the first line treatment options [2,3], since its resistance rates with regard to E. coli are low [21] and symptoms are treated effectively. For UTI, a single dose treatment has been shown to be efficient, serving as comparator in other studies as well [22-24].

Considering symptom relief as the most important factor for UTI patients [25], we chose ibuprofen for the intervention group. Ibuprofen is known to be efficient for pain reduction in many conditions. It is safe and efficient for pain control and shows anti-inflammatory activity.

### Outcomes

Studies on dose reduction or delayed treatment always have to address benefit and risk simultaneously. In this case, the benefit consists of a reduced number of antibiotic prescriptions, implying less resistance development and fewer side effects, whereas the potential risk of higher symptom burden has to be considered carefully. Therefore, we chose a combined primary endpoint with two components such as symptom course within 7 days and antibiotic usage up to day 28. Additionally, safety criteria will be assessed by several safety endpoints such as number of AE and SAE up to day 28.

### Safety

Treatment courses are very short in both groups. Empirically, adverse drug effects should occur less frequently with ibuprofen than with antibiotic therapy. Complicated disease courses are not expected, since UTI is a non-serious condition. The risk of pyelonephritis after non-antibiotic treatment of UTI is often mentioned, but little data exists. In one trial comparing antibiotic versus placebo treatment in patients with UTI 1 of 38 patients treated with placebo was suspected to have a pyelonephritis [12,26]. To avoid safety risks, study patients are instructed to reconsult in case of persistent/worsening symptoms. Patients with fever have to be reported immediately. To assess a long-term-follow up, telephone interviews will be conducted after 6 and 12 months.

## Conclusions

This trial may provide evidence for effectiveness for a new, non-antimicrobial treatment approach in general practice as indicated in the pilot study (HWI −01). If it could be shown that this strategy allows for a substantial reduction of antibiotics while being symptomatically effective and safe, treatment recommendations should be changed: anti-inflammatory agents as first choice therapy for uncomplicated UTI, and antibiotic treatment only for those who return with persisting or recurrent symptoms. Thus, the individual patient and society benefit from a reduced rate of antibiotic consumption reducing the pressure on resistance development.

## Abbreviations

AE, Adverse event; AIA, Activity Impairment Assessment; AUC, Area under Curve; CRF, Case report form; DDD, Daily defined dose; GP, General practice; ITT, Intention to treat; PP, Per protocol; RCT, Randomized clinical trial; SAE, Serious adverse event; UTI, Urinary tract infection.

## Competing interests

The authors have no conflicts of interest.

## Authors’ contributions

EHP and MMK had the original idea for the study. IG, JB and GS will supervise the practices and manage the trial on a day-to day basis. Data analysis will be performed by KW. All authors read and approved the final manuscript.

## Pre-publication history

The pre-publication history for this paper can be accessed here:

http://www.biomedcentral.com/1471-2334/12/146/prepub
